# An Overview of the TRP-Oxidative Stress Axis in Metabolic Syndrome: Insights for Novel Therapeutic Approaches

**DOI:** 10.3390/cells11081292

**Published:** 2022-04-11

**Authors:** Mizael C. Araújo, Suzany H. S. Soczek, Jaqueline P. Pontes, Leonardo A. C. Marques, Gabriela S. Santos, Gisele Simão, Laryssa R. Bueno, Daniele Maria-Ferreira, Marcelo N. Muscará, Elizabeth S. Fernandes

**Affiliations:** 1 Programa de Pós-Graduação, Universidade CEUMA, São Luís 65075-120, MA, Brazil; mizaelcalacioo@outlook.com (M.C.A.); gabyiisantos9@gmail.com (G.S.S.); 2 Instituto de Pesquisa Pelé Pequeno Príncipe, Curitiba 80250-060, PR, Brazil; suzanyhellen@gmail.com (S.H.S.S.); gisele_si@hotmail.com (G.S.); laryssaregis@hotmail.com (L.R.B.); daniele.ferreira@pelepequenoprincipe.org.br (D.M.-F.); 3 Programa de Pós-Graduação em Biotecnologia Aplicada à Saúde da Criança e do Adolescente, Faculdades Pequeno Príncipe, Curitiba 80230-020, PR, Brazil; 4 Programa de Pós-Graduação em Ciências da Saúde, Universidade Federal do Maranhão, São Luís 565085-080, MA, Brazil; jaquelinepessoasp@gmail.com; 5 Department of Pharmacology, Institute of Biomedical Sciences, University of São Paulo, São Paulo 05508-000, SP, Brazil; leomarques996@gmail.com (L.A.C.M.); muscara@usp.br (M.N.M.)

**Keywords:** TRP channels, metabolic syndrome, energy metabolism, hypoadiponectinemia, reactive oxygen species, inflammation

## Abstract

Metabolic syndrome (MS) is a complex pathology characterized by visceral adiposity, insulin resistance, arterial hypertension, and dyslipidaemia. It has become a global epidemic associated with increased consumption of high-calorie, low-fibre food and sedentary habits. Some of its underlying mechanisms have been identified, with hypoadiponectinemia, inflammation and oxidative stress as important factors for MS establishment and progression. Alterations in adipokine levels may favour glucotoxicity and lipotoxicity which, in turn, contribute to inflammation and cellular stress responses within the adipose, pancreatic and liver tissues, in addition to hepatic steatosis. The multiple mechanisms of MS make its clinical management difficult, involving both non-pharmacological and pharmacological interventions. Transient receptor potential (TRP) channels are non-selective calcium channels involved in a plethora of physiological events, including energy balance, inflammation and oxidative stress. Evidence from animal models of disease has contributed to identify their specific contributions to MS and may help to tailor clinical trials for the disease. In this context, the oxidative stress sensors TRPV1, TRPA1 and TRPC5, play major roles in regulating inflammatory responses, thermogenesis and energy expenditure. Here, the interplay between these TRP channels and oxidative stress in MS is discussed in the light of novel therapies to treat this syndrome.

## 1. Introduction

Metabolic syndrome (MS) is a complex pathology characterized by visceral adiposity, insulin resistance, arterial hypertension, and dyslipidaemia [1]. MS presents significant morbidity and mortality as it strongly increases the risk of developing different diseases, such as those affecting the cardiovascular system and type 2 diabetes (T2D) [2]. Its management is primarily aimed at reducing the risk for cardiovascular diseases (CVDs) and T2D, and includes lifestyle modifications and multiple drugs [3,4]. 

Abdominal obesity, insulin resistance and sedentary life-styles are major risk factors for MS [1]. These increase with ageing, by taking medicines which increase weight gain, by mitochondrial and endocrine dysfunctions, and genetic predisposition [3]. Although not the focus of the current review, the later findings on the genetic basis of MS have greatly contributed to further understanding the different underlying mechanisms and phenotypes of MS [5,6,7]. 

**Figure 1:**
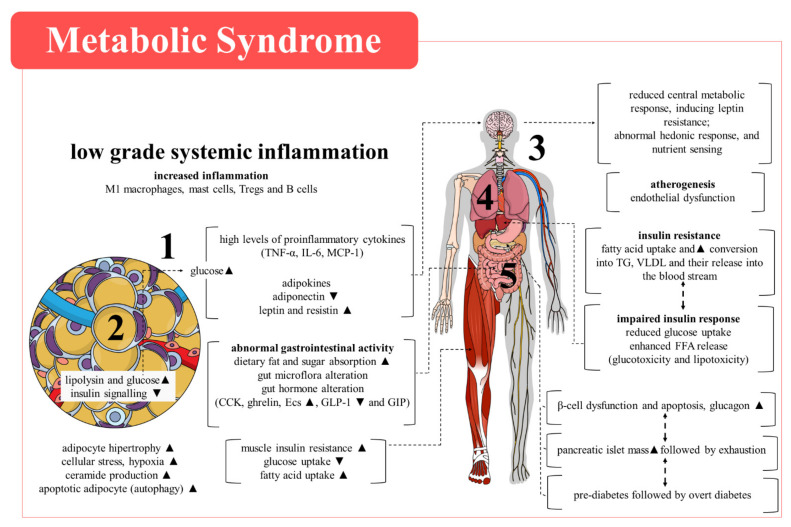
Mechanisms of metabolic syndrome (MS) pathophysiology. MS is a result of a metabolic imbalance which involves alterations in different tissues and a variety of molecules. (1) Insulin resistance is accompanied by (2) a low-grade inflammation in the adipose tissue characterized by reduction of adipokines such as adiponectin, enhanced levels of leptin and resistin, accumulation of inflammatory cells in the adipose tissue, paralleled with high levels of cytokines/chemokines and reactive oxygen species. Alterations of the central (hypothalamus and the brainstem) and peripheral mechanisms of hunger and satiety occur (3). All these events contribute towards (4) decreased energy expenditure, hyperglycaemia and dyslipidaemia, increasing the risk for type 2 diabetes and cardiovascular diseases. Nutrient absorption (5) and the gut microbiota play key roles in the modulation of MS, aiding the connection between the brain and metabolic tissues. TG—triglycerides; VLDL—very low-density lipoprotein; CCK—cholecystokinin; Ecs—estrogens; GLP-1—glucagon-like peptide-1; GIP—gastric inhibitor peptide; TNFα—tumour necrosis factor α; IL-6—interleukin-6; MCP-1—macrophage chemotatic protein-1.

Energy metabolism is influenced by an intricate network of molecules released and receptors expressed within metabolic organs such as the pancreas, liver, adipose tissue and skeletal muscle, connecting the periphery to the brain (Figure 1). Hypoadiponectinemia, inflammation and oxidative stress [8,9,10,11,12] account for some of the mechanisms involved in MS establishment and progression, with a clear interplay between them. Different pathways are suggested to modulate these mechanisms. In this context, members of the transient receptor potential (TRP) family of non-selective Ca^2+^ channels may play an important role in MS by regulating inflammatory responses, thermogenesis and energy expenditure [13,14,15,16]. Herein, we discuss the mechanisms of MS and the roles of TRPV1, TRPA1 and TRPC5, known as oxidative stress sensors and regulators of inflammation, in MS. We also present the clinical perspectives of targeting these receptors for MS management.

## 2. Adiponectin Dysregulation, Oxidative Stress and Inflammation as Mechanisms of Metabolic Syndrome

### 2.1. Adiponectin Dysregulation

Adiponectin is an adipokine secreted by adipocytes, first described in 1995 [17]. Although adiponectin functions were unknown at that time, by using mouse cells, this report was the first to demonstrate the existence of a link between insulin secretion, adipocyte differentiation and adiponectin release, and to suggest a role for this adipokine in the regulation of carbohydrate and lipid metabolism. In 1996, the human adiponectin was described in two different studies, which showed its presence in human adipose tissue and plasma samples [18,19].

In the last decades, it has become clear that adiponectin is an essential regulator of glucose and lipid metabolism and a great influencer of the risk for developing obesity, T2D, CVD and, therefore, for MS.

It is now known that adiponectin forms complexes of different molecular weights. Of note, the one of high molecular weight (HMW) was shown to be the most potent in reducing serum glucose levels in mice [20]. The same study demonstrated that the HMW complex is reduced in obese diabetic mice and that it becomes increased in both T2D mice and patients following treatment with rosiglitazone—a PPARγ agonist. Later, adiponectin-induced hypoglycaemia was found to be independent of insulin levels [21,22] but was able to improve insulin sensitivity [23]. Soon after, it was shown that adiponectin crosses the blood-brain barrier and induces the hypothalamic expression of the anorexigenic corticotrophin-releasing hormone (CRH), leading to weight loss and enhanced energy expenditure [23,24]. 

Adiponectin multimers can be cleaved in a fragment containing the C-terminal globular domain, which has potent effects on skeletal muscle cells. Full length adiponectin and its fragments may exert different actions on different cell types [25,26,27,28] by binding to the G-protein coupled adiponectin receptors type 1 (AdipoR1) and 2 (AdipoR2). AdipoR1 is constitutively expressed in every cell, especially in skeletal muscle, whilst AdipoR2 is greatly expressed in the liver [29]. They are both also expressed in various brain regions including hypothalamus, brainstem, hippocampus, and cortex [30]. 

A study by Bjursell and collaborators [31] investigated the contribution of AdipoR1 and AdipoR2 to energy metabolism homeostasis by using AdipoR1 and AdipoR2 knockout (KO) mice fed with a high-fat diet (HFD). They demonstrated that male AdipoR1KOs have greater adiposity and glucose intolerance, resulting in weight gain and energy expenditure, increased liver triglyceride (TG) and plasma leptin (a satiety hormone produced and secreted by white adipose tissue (WAT) [32]) levels, in addition to higher AdipoR2 mRNA expression in brown adipose tissue (BAT), a thermogenic tissue. On the other hand, the same study demonstrated that AdipoR2KOs are resistant to obesity, even eating more than control mice. The same KOs exhibited increased expression of CRH mRNA in the hypothalamus, less plasma leptin and cholesterol, lower liver TG, greater plasma and adiponectin levels, and higher glucose tolerance and energy expenditure. Interestingly, AdipoR2KOs presented with decreased levels of AdipoR1 mRNA in the liver and BAT [31]. 

Similar to AdipoR1KOs, mice with adiponectin gene ablation fed with a high-fat/high-sucrose diet had severe insulin resistance [33]. Interestingly, mice lacking adiponectin fed with a normal diet presented delayed free-fatty acid (FFA) clearance, and higher plasma and adipose tissue tumour necrosis factor-α (TNFα) levels. Injection of a full-length adiponectin producer adenovirus reversed this phenotype in adiponectin KO mice. 

**Figure 2:**
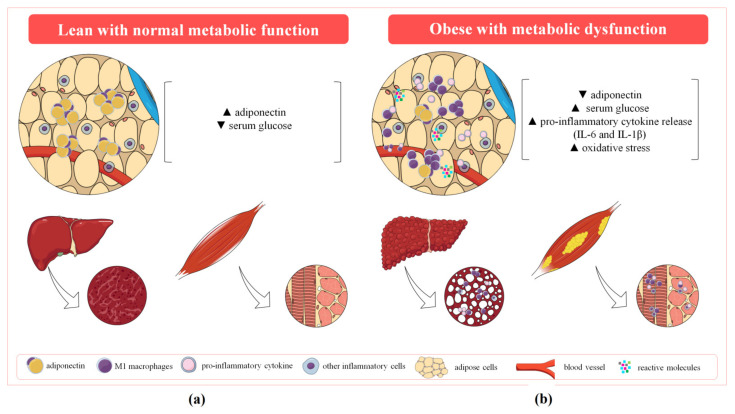
Fat tissue inflammation and adiponectin dysregulation in metabolic syndrome. (**a**) In lean individuals, adipose tissue contains few M2 macrophages and adipocytes produce high levels of adiponectin. Their insulin levels and sensitivity are regulated and result in normal glucose levels. (**b**) Individuals with metabolic dysfunction present with inflamed metabolic tissues with fat deposition and ROS production, which result in reduced cell viability and insulin resistance/high glucose levels.

Human studies have associated hypoadiponectinemia (Figure 2), with excessive intra-abdominal fat and multiple defects in glucose and energy metabolism in MS. The syndrome has also been linked to increased circulating levels of cytokines (e.g., interleukin (IL)-6 and IL-1β) and soluble adhesion molecules (e.g., P-selectin and ICAM) [34]. The same study suggested that low adiponectin production is an underlying cause of endothelial damage and low-grade systemic inflammation in MS. The data are supported by previous studies showing that hypoadiponectinemia increases the risk for coronary artery disease (CAD) in men [35], and data from mice that showed that adiponectin protects against vascular damage following mechanical injury [36]. In addition, obese patients with CAD have diminished plasma levels of adiponectin and lower expression of adiponectin receptors in peripheral monocytes in comparison with those without CAD, while macrophages from CAD patients present impaired release of IL-10 following adiponectin incubation [37].

Oxidative stress has been suggested as a cause of hypoadiponectinemia [38,39,40]. Indeed, exposure of pre-adipocytes (3T3-L1 cells) to oxidants such as hydrogen peroxide (H_2_O_2_), glucose oxidase or 4-hydroxynonenal (4-HNE), results in decreased expression and secretion of adiponectin. The low levels of this adipokine caused by oxidants are accompanied by increased production of pro-inflammatory cytokines (TNFα, IL-6) and chemokines (macrophage inflammatory protein-1; MCP-1) by adipocytes [40,41,42,43]. The contribution of oxidative stress to MS is discussed below.

### 2.2. Oxidative Stress

Oxidative stress is defined as the imbalance between the production and neutralizing pathways of reactive oxygen and reactive nitrogen-derived pro-oxidant species (ROS and RNS, respectively) in favour of these species. The link between oxidative stress and inflammation pathways highlights the burden of this condition in MS [44].

The physiological production of ROS (such as superoxide anion—O_2_^−^, hydroxyl radical—OH, H_2_O_2_ and hypochlorous acid—HClO) and RNS (such as nitric oxide—NO and peroxynitrite anion—ONOO^−^) occurs via different endogenous enzymatic pathways (e.g., nicotinamide adenine dinucleotide phosphate oxidases—NOX, NO synthases—NOS, myeloperoxidase—MPO, xanthine oxidase—XOs, amongst others.). Although ROS and RNS signalling contribute to diverse cellular processes [45,46], under oxidative stress, there is increased availability of these species leading to harmful effects in various disease states, including in MS. For example, O_2_^−^ produced by NOX activates XOs inducing tetrahydrobiopterin (BH4) oxidation, endothelial NOS uncoupling and the consequent lowering of NO production and bioavailability, an essential hallmark of the pathogenesis of T2D and hypertension [47]. Other cellular effects of oxidative stress involve damage to proteins, membrane lipids, and nucleic acids. OH.—induced lipoperoxidation and DNA damage (as assessed by the formation of 8-hydroxy-2′-deoxyguanosine-8-OHdG), are well-established markers of chronic inflammation in MS [48]. 

On the other hand, neutralizing antioxidant pathways mitigate the reactivity of ROS [49]. Primary antioxidant pathways include superoxide dismutase (SOD), glutathione peroxidase (GPx), and catalase. Additional pathways include glutathione reductase, thioredoxin (TRX), and glutaredoxin. Other non-enzymatic pathways comprise reduced glutathione (GSH), bilirubin, and low molecular weight compounds of dietary origin (e.g., vitamins A, C, E, flavonoids, zinc, and selenium) [48]. In this way, antioxidant pathways provide conditions for controlled production of physiologically relevant oxidant species (such as H_2_O_2_) and the maintenance of homeostasis.

Oxidative stress has also been directly linked to the pathogenesis of CVDs such as hypertension, and insulin resistance in T2D [50]. In MS, oxidative stress is mainly characterized by the diminished expression and activity of antioxidant pathways secondary to a decrease in the levels of nuclear factor E2-related factor 2 (NRF2), in plasma samples from patients with MS [51]. These findings were also present in experimental models of obesity [52].

Hypertension is associated with reduced bioavailability of NO and increased ROS production either from dysfunctional mitochondrial or enhanced NOX expression in endothelial cells. The peroxynitrite anion, the product of the reaction between O_2_^−^ and NO, also contributes to dysfunctional systemic vessel tonus control [53]. Furthermore, dyslipidaemia, insulin resistance, hyperglycaemia, and other factors, contribute to mitochondrial dysfunction and enhance mitochondrial O_2_^−^ production by endothelial cells, cardiomyocytes and pancreatic β-cells [54], the latter being particularly vulnerable to oxidative stress due to the low expression of antioxidant defences in these cells. 

Diverse mechanisms are related to the effects of oxidative stress on pancreatic β-cell function, including altered expression of micro-RNAs responsible for the gene regulation of redox signalling pathways [55]. Excessive ROS production, together with hyperglycaemia, contributes to glyceraldehyde-3-phosphate dehydrogenase inhibition, which results in the accumulation of glycolytic pathway precursors (such as fructose-6-phosphate and glyceraldehyde-3-phosphate). In this case, the subsequent activation of polyol cascades (by advanced glycation end-products—AGEs) causes NADPH depletion and reduced bioavailability of GSH [56]. In this case, the activation of nuclear factor-κB (NF-κB) and NOX promotes oxidative stress and a pro-inflammatory status that contribute to the vascular complications of T2D due to the low NO bioavailability and high expression of cell adhesion molecules [57]. In addition, the enhanced vasoconstriction elicited by endothelin-1 and other endogenous vasoconstrictors (such as prostaglandin H_2_ and thromboxane A_2_) impairs both the endothelial function and vascular wall integrity, particularly at the microcirculation level [58]. Therefore, AGEs play an important role in the delicate interface between T2D and CVDs [59].

As previously discussed, oxidative stress (Figure 2) can contribute to hypoadiponectinemia and inflammation, and thus, to obesity. Obesity is characterized by a systemic pro-inflammatory status, mainly due to the development of insulin resistance [60]. From a cellular perspective, impaired mitochondrial function and biogenesis caused by either hyperglycaemia and/or hyperlipidaemia impairs the insulin signalling pathway [61]. This, in addition to dysfunctional adipose tissue, enhances oxidative stress by activating pro-inflammatory pathways in adipocytes [62], which are amongst the aetiological factors of T2D and CVDs [63]. 

Interestingly, increased circulating levels of TRX are present in T2D patients [64] and have been associated with higher risk for CVD in individuals with MS [65]. 

### 2.3. Inflammation

In addition to adiponectin dysregulation and oxidative stress (Figure 2), studies have provided compelling evidence that the progress of metabolic dysfunction is closely related to a state of low-grade chronic inflammation [66,67,68], which is primarily characterized by recruitment of pro-inflammatory macrophages to the adipose tissue. Macrophages enhance the inflammatory response [69,70], contributing to the accumulation of ectopic lipids and the development of insulin resistance [71]. M1 and M2 macrophages play an important role in adipose tissue during low-grade inflammation [72] through the production and release of TNFα, IL-1β, and IL-6, and IL-10, respectively [73,74]. M1 macrophages recruited to the pancreatic islets cause pancreatic β-cell dysfunction and apoptosis [74]. Furthermore, the production of pro-inflammatory cytokines within the adipose tissue leads to adipocyte hypertrophy [70]. The local release of FFA, especially saturated fatty acids, activates toll-like receptor 4 on macrophages [75,76], triggering the activation of NF-κB and the additional expression of pro-inflammatory cytokines [77]. These events continuously contribute to insulin resistance in the adipose tissue, liver, and skeletal muscle [78,79]. The above data reinforce the importance of inflammatory imbalance to the adipose tissue changes in MS and its comorbidities/complications [67]. Indeed, adipose tissue inflammation also impacts other tissues and organs such as the liver [80,81], pancreas [82] and muscles [83]. Fat deposition in these organs is particularly deleterious [10]. 

Liver resident macrophages (Kupffer cells) can also be polarized into M1 and produce TNFα as a result of a lipid-rich diet that, in turn, contributes to increased glucose release by gluconeogenesis, lipid production and storage by inhibiting intracellular lipases [74]. The metabolic complications associated with a decline in insulin release lead to glucolipotoxicity in the pancreatic islets and the adipose tissue [84,85], imbalance of redox states, and mitochondrial dysfunction [85]. An obesogenic diet promotes endoplasmic reticulum stress and pancreatic β-cell dysfunction, with consequent reduction of insulin production [84]. These alterations are closely associated with increased inflammation, oxidative stress, and subsequent damage to DNA, proteins, cellular lipid, and potentially cell death [86]. In fact, liver damage may be driven by the secretion of pro-inflammatory cytokines (e.g., TNFα) [87], hypoadiponectinemia [88,89], and high levels of resistin and leptin [80,90,91] in the adipose tissue. Increased hepatic lipid accumulation into the liver followed by de novo lipogenesis and reduction of fatty acid oxidation [92,93] leads to histological damage characterized as simple steatosis, non-alcoholic steatohepatitis, or cirrhosis, and even hepatocellular carcinoma in more serious cases [94,95].

Although the studies are still controversial [96,97,98,99], pancreatic fat may be associated with β-cell dysfunction and insulin resistance [100,101]. Importantly, sarcopenic obesity is directly related to additional weight gain [102] and poor physical function and ability [103]. 

In addition to macrophages, T cells also play a role in MS. Mice fed HFD present with higher numbers of CD8^+^ and smaller populations of CD4^+^ and regulatory T cells in the epididymal WAT in comparison with normal chow-fed mice [104]. CD8^+^ cell influx precedes that of M1 macrophages in the adipose tissue, increasing inflammation and systemic insulin resistance. In agreement, mice lacking T cells are protected against obesity-induced T2D in HDF-fed mice, which is associated with less macrophage accumulation and down-regulation of inflammatory cytokines/chemokines (MCP-1, RANTES, IL-6, TNFα and IFNγ) in skeletal muscle and adipose tissue samples [105]. Conversely, in another report, T cell recruitment and IFNγ up-regulation occurred in epididymal WAT following macrophage influx [106]. Overall, these data show the contribution of Th1 cells to adipose tissue inflammation in MS. Additionally, Th17 cells contribute towards a pro-inflammatory phenotype in the adipose tissue and insulin resistance [107], whilst Th2 cells are suggested to protect against obesity [108]. Interestingly, the percentage of Th2 cells in human adipose tissue samples negatively correlates with systemic inflammation and insulin resistance [108]. 

## 3. Transient Receptor Potential Channels

### 3.1. General Overview of TRPV1, TRPA1 and TRPC5 Channels

TRP channels are polymodal cation channels that mediate Ca^2+^ influxes across the cell membrane [109]. Cationic influxes through TRPs depolarize the cell membrane and activate many cellular responses. The development of agonists, antagonists and KO mice for TRPs has helped to define their expression sites and pathophysiological functions throughout the last few decades. Although TRPs have different expression patterns, their wide physiological distribution indicates their involvement with biological processes in different cells, tissues, and organs [110,111,112]. The mammalian TRP family is composed of 28 members classified into six sub-families: vanilloid (TRPV), ankyrin (TRPA), canonical (TRPC), melastatin (TRPM), mucolipin (TRPML), and polycystin (TRPP) [113,114]. 

TRPs are expressed in both neuronal and non-neuronal cells and mediate a range of responses including nociception, inflammation, vascular tonus, cell contractility, energy expenditure, amongst others. These channels can be activated by a plethora of endogenous stimuli such as inflammatory mediators, lipids and oxidative/nitrosative stress products. As the focus of this review is to discuss the TRP-oxidative stress axis in different metabolic tissues in MS, the roles of TRPV1, TRPA1 and TRPC5 are presented. 

**Figure 3:**
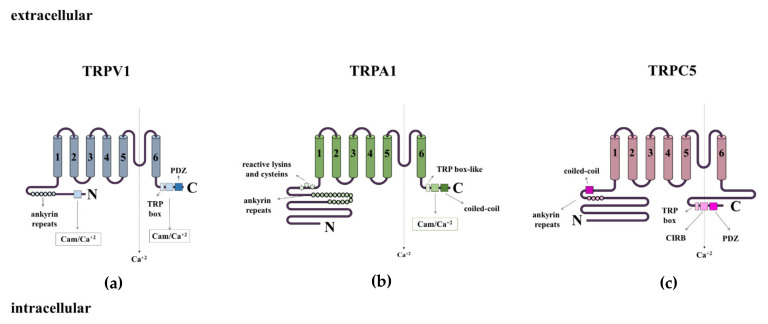
TRPV1, TRPA1 and TRPC5 structures. (**a**) TRPV1, (**b**) TRPA1 and (**c**) TRPC5 structures are composed of different domains including six transmembrane domains with a pore region, *N* and C-terminus, ankyrin repeats, coiled-coil, calmodulin (CaM)/Ca^2+^-binding region, TRP-box, calmodulin (CaM)/inositol 1,4,5-trisphosphate (IP_3_) receptor binding (CIRB), and PDZ domains.

TRPV1 (Figure 3a) was the first to be described and is the most extensively studied member of the TRP family [115,116]. It contains six transmembrane domains or sub-units (S1–S6) and a hydrophobic pore region between S5 and S6, in addition to intracellular domains—a long *N*-terminus with multiple ankyrin repeats and a short C-terminal region [115]. These domains are now known to be essential as protein and compound-binding sites and, therefore, detrimental to the modulation of TRPV1 functions. Details on TRPV1 binding sites have been recently revised [117]. Fatty acid-derived products such as hydroxyeicosapentaenoic acid (12 (S)-HPETE) [118], 20-hydroxyeicosatetraenoic acid (20-HETE) [119], 9- and 13-hydroxyoctadecadienoic acids (9-HODE and 13-HODE) and oxidized forms [120], endocannabinoids such as anandamide [121], hydrogen sulphide (H_2_S; [122]), and ROS (H_2_O_2_; [123]), amongst others, are able to endogenously activate the receptor, either directly or by sensitization. TRPV1 is widely expressed in neurones and also in metabolic tissues including the adipose [124,125] and liver tissues [126,127]. TRPV1 is also expressed in M1 macrophages [127,128] and T cells [129,130,131], already discussed herein, as a key inflammatory factor of in MS. On the other hand, TRPV1 expression in pancreatic β-cells is controversial [132,133]. 

TRPA1 (Figure 3b) also consists of six sub-units (S1–S6) and a hydrophobic pore region between S5 and S6 and has large intracellular *N* and C-terminal domains. A domain containing five ankyrin repeats surrounds the coiled-coil region [134]. Key cysteines necessary to channel activation by electrophiles are found within the pre-S1 region [134]. TRPA1 is broadly expressed throughout the body including in metabolic tissues and cells [135,136,137]. TRPA1 can be activated by a variety of molecules produced and released during oxidative phosphorylation, including methylglyoxal [138], 4-HNE, 15-deoxy-delta(12,14)-prostaglandin J_2_ (15d-PGJ2) and H_2_O_2_ [139]. These molecules, and TRPA1, have been associated with anti-hyperglycaemic and anti-obesity effects which are further discussed herein. 

TRPC5 (Figure 3c) is formed by a four-fold symmetric homotetramer, and each of the four monomers presents with a compact cytosolic domain and a transmembrane domain. The cytosolic domain is composed of the *N*-terminal region with an ankyrin domain and a region of seven α helices, whilst the C-terminal sub-domain contains a connecting helix and a coiled-coil domain. The transmembrane domain contains sub-units (S1–S6), a TRP domain, and several small helices, including a pore helix [140]. The presence of a disulphide bond at the extracellular side of the pore and a preceding small loop confer functionality to TRPC5 [140]. Of importance, as previously demonstrated for TRPV1 and TRPA1, which are able to functionally interact as dimmers (recently revised [141]), TRPC5 can also form functional homo and heterocomplexes with other receptors of the same family, such as TRPC4 and TRPC1 [142,143] that exert different functions, from inflammation to vascular remodelling. TRPC5 complexes are widely expressed in the central nervous system (CNS) and at lower levels in other tissues and cells [144]. In the context of MS, TRPC5 has an important role connecting metabolic tissues and the brain. TRPC5 can be activated by a range of molecules including H_2_O_2_ [145], reduced TRX [146], and fatty acids [147]. 

The roles of TRPV1, TRPA1 and TRPC5 as mediators of oxidative stress and inflammation and, as modulators of MS are discussed below. 

### 3.2. TRPs as Key Sensors of Oxidative Stress

TRP channels play essential roles in cellular function and disease [148]. Interestingly, specific TRPs are activated by ROS, amongst the several stimuli described to date. TRPM2 was the first TRP channel described as sensitive to ROS [149]. It is now known that TRPV1, TRPA1 and TRPC5 are not only oxidative stress sensors but also modulate oxidative stress pathways. 

Reactive molecules, such as those involved in oxidative stress, are able to either directly activate or sensitize the TRP channels discussed herein. Evidence for the functional activation of these receptors by reactive molecules is listed in the Table 1. Different studies have demonstrated the ability of H_2_O_2_ to sensitize TRPV1 [123,150,151,152]. An initial report showed that H_2_O_2_ potentiates heat-induced membrane currents mediated by TRPV1 in HEK293T cells [150]. Next, this ROS was found to cause thermal hyperalgesia by TRPV1-dependent and independent mechanisms when intra-articularly injected in mice [151], and to potentiate apnoeic responses in rats by acting on both TRPV1 and TRPA1 when given as an aerosol [153]. H_2_O_2_ also induced increases in coronary blood flow, a response partially mediated by TRPV1 [123]. In the same study, H_2_O_2_ promoted the activation of intrinsic TRPV1-specific currents in isolated mouse coronary endothelial cells, which were blunted in endothelial cells lacking TRPV1. Interestingly, the prolonged exposure of TRPV1 to H_2_O_2_ and reactive aldehydes, such as 4-HNE, impairs TRPV1 functions contributing to microvascular dysfunction in T2D [123,154]. 4-HNE-induced inhibition of TRPV1-mediated responses in coronary arterioles was suggested to be due to direct binding of this aldehyde to the channel [154]. H_2_O_2_ and O_2_^−^ generation can be modulated by TRPV1, indicating a feedback loop between this channel and ROS production [155,156]. 

As for TRPV1, TRPA1 is a well-documented oxidative stress sensor. The first evidence that the channel could be activated by reactive molecules demonstrated the ability of 4-HNE to evoke pain via TRPA1 activation on rodent nociceptive neurones. This event led to the release of substance P, causing neurogenic inflammation [157], and it was supported by further evidence [160]. In vivo and in vitro models showed that H_2_O_2_ triggers the neuronal activation of TRPA1 [160,163]. Oxidative stress can also activate TRPA1 on non-neuronal tissues and cells. For instance, 4-HNE induces the dilation of cerebral arteries [164] and increases of Ca^2+^ influx in pancreatic β-cells [137] following activation of this channel. Neurogenic vasodilatation is also mediated by TRPA1, a response which requires peroxynitrite generation [165].

TRPC5 is perhaps one of the most interesting TRP members with respect to oxidative stress signalling. TRPC5 can be activated by both oxidant (H_2_O_2_; [145]) and antioxidant (reduced TRX; [146]) molecules; the latter shown to be a response dependent on TRPC1/TRPC5 complexes in non-neuronal cells. Interestingly, a recent report showed that eNOS-derived NO causes suppression of TRPC5 activity in endothelial cells [166]. Considering the reduced activity of eNOS and NO bioavailability in MS, it is possible that TRPC5 function in this syndrome is linked to regulation of blood vessel tonus and pressure control. TRPC5 is constitutively expressed in the brain and in metabolic tissues such as the adipose. Its contribution to energy metabolism is further discussed in this review. 

Amongst the many cellular responses mediated by TRPs, the regulation and maintenance of inflammatory mechanisms have unique roles, given TRP permeability to Ca^2+^, which mediates transcription, translation, cellular division and apoptosis. In this way, ROS-based signalling mechanisms via TRPs are critical points worthy of deeper investigations in MS [48]. Different from TRPM2-redox activation by OH^.^ (mainly produced in the Fenton reaction of iron-catalysed H_2_O_2_ decomposition), the main mechanism of TRPA1, TRPV1 and TRPC5 activation by ROS is a redox-sensitive pathway via cysteine disulphide formation from proximal cysteine residues [167]. In fact, at least four cysteine residues have been described in the redox-sensitive mechanism of TRPA1 activation: Cys-421, Cys-621, Cys-641 and Cys-665 [158,163,168]. Cys-621 is the binding residue for 4-HNE in TRPV1 [154] and Cys-158 the binding residue for H_2_O_2_ in the same channel [152].

### 3.3. TRPs as Regulators of Inflammation

The roles of TRPV1, TRPA1 and TRPC5 in inflammation have been widely investigated in past years. Different pieces of evidence indicate these channels participate in inflammatory events including cell migration, inflammatory mediator release and cell survival. Since both macrophages and T cells play a role in MS, this session focuses on the impact of these channels on T cell and macrophage responses. 

The first indication that these channels are functional in inflammatory cells dates from the late 1990s. Incubation of capsaicin with activated human T cells caused Ca^2+^ mobilization [129]. TRPV1 expression in mouse CD4^+^ T cells was later confirmed and shown to mediate the production of different cytokines (IL-4, IL-5, IL-6, and IL-17), associated with increased phosphorylation of kinases and NF-κB [130]. These findings were supported by data from Jurkat T cells following treatment with the TRPV1 inhibitor BCTC, and from TRPV1KO mice sensitized with ovalbumin, as both the inhibitor-treated cells and the animals with gene ablation of the channel resulted in less cytokines [130,131]. TRPV1 expression was also confirmed in mouse CD11c^+^ dendritic cells and CD11b^+^F4/80^+^ macrophages [169]. Increased channel activation promoted higher secretion of cytokines (higher level of IL-6, IL-1β, TNFα, and IL-23) by dendritic cells [169]. TRPV1 was found to regulate macrophage and monocyte responses. In the absence of TRPV1, mouse macrophages are more susceptible to apoptosis, have impaired ability to perform phagocytosis and to produce ROS and NO, and release high levels of cytokines during bacteraemia associated with worsening of the disease in vivo [156]. In mouse cerebral malaria, the lack of TRPV1 triggers less cerebral swelling, increased oxidative stress, and diminished production of cytokines [170]. These results indicate that, depending on the stimuli, the modulation of inflammation by TRPV1 can result in either protection against, or damage, in diseases. In fact, TRPV1 is highly expressed in M1 macrophages and its activation in these cells leads to inhibition of M1 polarization [128,171]. The inflammatory response is not modulated by TRPV1 only in microbial infections, but also in many different chronic diseases such as rheumatoid arthritis [172], colitis [169], rhinitis [130], and MS [173,174]. 

Human circulating leukocytes, Jukart T and mouse CD4^+^ T cells also express functional TRPA1 [175,176,177]. By using TRPA1 antagonists and KO mice, conflicting results have been found concerning the channel role in immune cells. Pre-treatment of murine splenic T cells with TRPA1 antagonists (A967079 and HC-030031) abolished T cell receptor-induced Ca^2+^ currents, as well as reduced T cell activation and cytokine release (TNFα, IFNγ and IL-2) by these cells [178]. Another report showed however, that TRPA1KO CD4^+^ splenic T cells present enhanced and prolonged T cell receptor-induced Ca^2+^ currents [176]. A compensatory role via TRPV1 was found to be involved in this response. In addition to T cells, monocytes and macrophages also express TRPA1. TRPA1 activation in cultured primary human monocytes triggers TNFα release and impairment of IL-10 production [179]. THP-1-derived macrophages express functional TRPA1 [179,180]. In these cells, TRPA1 was found to mediate the effects of lysophosphatidylcholine (an atherogenic lipid; [181]) on mitochondrial ROS production and membrane depolarization, IL-1β production and cell survival [180]. TRPA1 is also involved in the ATP actions on macrophages, contributing to mitochondrial damage, IL-1β secretion, and cell death [179]. Of note, ATP is an important molecule in atherosclerosis and hypertension [182,183]. These results infer that TRPA1 can contribute towards CVD in MS by regulating macrophage-mediated responses. Analysis of mouse atherosclerotic aortas indicated they express higher TRPA1 levels than control samples, especially in macrophages found in the atherosclerotic lesions [184]. TRPA1 blockade by HC-030031 or its genetic ablation resulted in larger lesions, hyperlipidaemia, and increased levels of pro-inflammatory mediators in the aorta (TNFα, IL-6, MCP-1 and MIP-2). The same study demonstrated that oxidized low-density lipoprotein directly activates TRPA1 and that channel activation protects against the formation of foam cells by reducing lipid accumulation [184]. These studies highlight a dual role (protective or deleterious) for TRPA1 in atherosclerosis. 

In an initial report, TRPC5 was found to be expressed at very low levels in murine resting effector T lymphocytes, and to become up-regulated following activation of these cells [185]. TRPC5 mediated Ca^2+^ currents induced by the lectin galectin-1 produced by regulatory T cells; this response was abrogated by the TRP blocker SK&F96365 and receptor knockdown, and also in T cells from TRPC5KO mice. The same study showed that TRPC1 but not TRPC4 is detected in T cells. This was the first evidence that the activation of TRPC5 complexes can contribute to autoimmune suppression. In macrophages (RAW 264.7 cells), TRPC5 inhibition by antagonism with ML-204 or RNA silencing, caused cell polarization to a M1 phenotype which was characterized by increased secretion of pro-inflammatory cytokines involving NF-κB activation (TNFα, IL-1β and IL-6) [186]. TRPC5KO mice fed HFD had higher numbers of M1 macrophages infiltrating their aorta and greater serum levels of TNFα and IL-6 [186]. In addition, TRPC5 deletion or antagonism by ML-204 restored phagocytosis in macrophages challenged with LPS and bacterial TRX [187]. These pieces of evidence indicate a protective role for TRPC5 in inflammation and CVD. 

The above findings highlight the importance of TRPV1, TRPA1 and TRPC5 as modulators of inflammation in MS and are supported by studies performed with their endogenous agonists, including H_2_O_2_, 4-HNE and reduced TRX. H_2_O_2_ is suggested to act as a first messenger for different pro-inflammatory ligands including NO and AGEs [188], in addition to its role as second messenger in intracellular pathways which lead to the expression of pro-inflammatory mediators via redox-sensitive kinases and NF-κB activation [189,190,191]. A rapid increase of H_2_O_2_ following tissue damage also triggers fast leukocyte recruitment [192]. 4-HNE induces ciclooxigenase-2 expression in RAW 264.7 and peritoneal macrophages, in addition to leukocyte migration in mice, and via kinase activation [193]; these effects may contribute to the pro-inflammatory roles of prostaglandins. In accordance, 4-HNE activates NF-κB in vascular smooth muscle cells and 5-lipoxygenase production in murine macrophages [194,195]. Conversely, 4-HNE can cause inhibition of NF-κB activation as observed in monocytes, Jukart T and rat kupffer cells treated with the aldehyde [195,196,197]. These findings suggest that 4-HNE can be either pro or anti-inflammatory depending on the cell/tissue. An anti-inflammatory role has been attributed to TRX. Indeed, in vitro incubation of TRX-1 induces a M2 macrophage phenotype, and also reduces TNFα and MCP-1 generation by M1 macrophages [198]. The same study showed that TRX protects against atherosclerosis by shifting macrophage polarization to M2 in ApoE2.K1 mice with severe atherosclerotic lesions. The TRX-1-mimetic peptide CB3 reduced ROS production and NF-κB-mediated release of cytokines/chemokines (IL-1, IL-6, IL-1β and MCP-1) by cultured macrophages [199]. CB3 also presented atheroprotective effects in ApoE2.Ki mice fed HFD, which was associated with reduced levels of pro-inflammatory cytokines, increased production of anti-inflammatory proteins (adiponectin and IL-10) in the plasma, and a M2 macrophage phenotype in aortic lesions.

## 4. The Roles of TRPV1, TRPA1 and TRPC5 in MS

**Figure 4:**
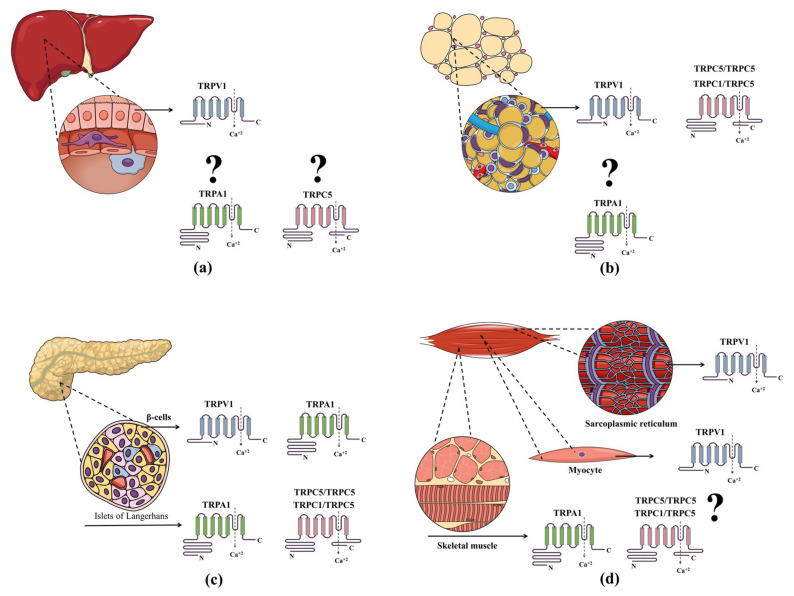
TRPV1, TRPA1 and TRPC5 expressions in metabolic tissues. (**a**) Liver, (**b**) adipose tissue, (**c**) pancreas and (**d**) skeletal muscle. All these TRPs are detected in the pancreas either as transcripts or functional proteins. TRPV1 is found in all metabolic tissues (liver, adipose tissue, pancreas and skeletal muscle). Additionally, TRPA1 and TRPC5 (either as homo or heterodimers) are expressed in the skeletal muscle and adipose tissue, respectively. The question tag (**?**) represents expressions yet to be confirmed: TRPA1 in the liver and adipose tissues, and TRPC5 in the skeletal muscle.

This section presents current data on the expression patterns (Figure 4; Table 2) and roles of TRPV1, TRPA1 and TRPC5 in the regulation of metabolic tissues, as well as in the connection between these tissues and the brain. Importantly, the combined expression of all the TRPs discussed herein contributes to regulate the functions of metabolic tissues and cells. 

### 4.1. Regulation of Insulin and Insulin Resistance

The involvement of TRPs in insulin resistance first remits to TRPC4 and TRPM2, as their activation leads to pancreatic cell depolarization and Ca^2+^ influx, thus regulating insulin secretion by distinct mechanisms [212,213]. However, the systemic expression of other TRPs may be altered by hyperglycaemia, concomitantly connecting T2D and CVDs in MS [214]. 

In addition to the controversial data regarding TRPV1 expression in pancreatic β-cells [132,133], its contribution to insulin resistance is unclear. Mice prone to diabetes lacking pancreatic innervations are protected from the development of insulitis and pancreatic disease; these data lead to the conclusion that TRPV1 activation is associated with the pathogenesis of type-1 diabetes [215]. Furthermore, TRPV1KO mice had a longer life-span than wild-type (WT) animals, in addition to higher insulin sensitivity [216]. In agreement, both the chemo-denervation of TRPV1 neurones and its blockade induced glucose-dependent insulin secretion in rodents [172,217,218]. These findings suggest the involvement of neuronal TRPV1 activation in insulin resistance and islet inflammation. In contrast, other studies showed that TRPV1KO mice fed with HFD present with higher insulin resistance than WTs under the same dietary conditions [219]. Interestingly, the intake of low doses of dietary capsaicin, a TRPV1 activator, has been associated with improved clinical signs in obesity and T2D [220,221]. 

Pancreatic β-cells and other insulin-secreting cells express TRPA1 [133,137], after which activation of glucose-dependent insulin secretion by these cells is potentiated [137]. In vivo studies showed that the metabolic activity of TRPA1 involves glucose uptake stimulation, intestinal incretin hormone secretion, and inhibition of food intake [222,223,224]. It was also indicated that TRPA1 agonists such as cinnamaldehyde improve diabetes in vivo through glucose transporter (GLUT4) translocation in peripheral tissues [222]. Recently, it was demonstrated that the effects of endogenous catechol oestrogens on insulin secretion by pancreatic β-cells is mediated by TRPA1 activation, thus making of this receptor a link between oestrogen metabolism and metabolic diseases [225].

Additional experimental data showed reduced TRPA1 expression in the islets of Langerhans obtained from rodents with T2D [206]. However, another study demonstrated that the deleterious effects of streptozotocin (a compound used for experimental diabetes induction) on β-cells are independent of TRPA1 activation [226]. In a model of chronic pancreatitis (induced by the injection of trinitrobenzene sulfonic acid), it was demonstrated the involvement of TRPA1 in the development of this condition, as TRPA1KOs showed reduced pancreatic inflammation in comparison with WT mice [227]. In contrast, allyl isothiocyanate (a TRPA1 agonist) was able to enhance insulin sensitivity and glucose tolerance in mice fed HFD, and the effects are most probably related to the reversal of the impaired mitochondrial function [228]. Studies with endogenous activators of TRPA1 such as 4-HNE, further support the involvement of TRPA1 in the modulation of glucose levels and insulin resistance. Treatment of gastrocnemius muscle and L6 muscle cells with 4-HNE reduced insulin signalling and insulin-induced glucose uptake in skeletal muscle cells by increasing oxidative stress and depletion of GSH [229]. In addition, this aldehyde was negatively correlated with insulin sensitivity in obese subjects [230]. In this context, the complex role of TRPA1 in insulin resistance suggests that the regulation of TRPA1 activation could be a novel therapeutic strategy, although additional studies are needed to properly elucidate this pathway in MS. 

There is little data on the pancreatic expression of TRPC5 [208] and no reports so far on the role of this receptor in insulin resistance. Despite that, TRPC1 can form complexes with TRPC5 [142], and there is growing evidence on the pancreatic expression of TRPC1 [208,231,232] as well as on its function as regulator of glucose tolerance and insulin secretion [233,234]. The existence of a TRPC5-aerobic glycolysis axis was also observed in colorectal cancer cells [235]. In addition, TRPC5 was found to mediate neuronal cell damage and death under metabolic stress such as oxygen-glucose deprivation [236]. Therefore, it is expected that further developments in the field will be able to overrule or demonstrate the importance of TRPC5 complexes in insulin resistance and/or their roles as sensors of glucose levels.

### 4.2. Regulation of Adypocytes

The adipose tissue plays an essential role in MS by influencing glucose and lipid balances. There are different types of adipose tissue (white, brown, and beige), and their cellular content, secreted substances and location determine MS development and progression. It is important to highlight that WAT stores excess energy as TGs, whilst the BAT is involved in energy expenditure. The differentiation of WAT into a BAT-like phenotype is known as browning of WAT and is characterized by thermogenic beige adipocytes also called “brite” cells. BAT and beige adipocytes contribute to reduction of insulin secretion and, therefore, to control T2D, in addition to obesity. These aspects have been recently reviewed [10,237,238].

TRPV1 expression was shown in cultured 3T3-L1-preadipocytes and in mouse and human adipose tissue samples [124,125,200,201]. The first study, in 2007 [125], demonstrated that TRPV1 is down-regulated during adipogenesis, and that capsaicin incubation prevents this response in 3T3-L1 cells, indicated by reduced TG content, lower expressions of PPAR-γ and fatty acid synthase; capasaicin effects in adipogenesis were blunted by TRPV1 knockdown. TRPV1 expression was also decreased in the visceral adipose tissue of obese mice and in the visceral and subcutaneous fat of obese patients in comparison with lean controls [125]. Dietary capsaicin stimulates the expression of the BAT-specific thermogenic uncoupling protein-1 (UCP-1) and the browning of WAT in WT but not TRPV1KO mice, by increasing the expression of sirtuin-1 [201]. In turn, the deacetylation of PPARγ occurs leading to reduced lipid synthesis and obesity [201]. A similar effect was seen for another TRPV1 agonist, monoacylglycerol, shown to increase UCP-1 expression and to impair the accumulation of visceral fat in high fat/high sucrose diet-fed mice [239]. The role of TRPV1 as a thermogenic receptor in adipocytes was also confirmed by a recent study in which TRPV1+-thermogenic adipocyte progenitors were characterized [240]. 

Despite consuming equivalent energy and absorbing similar quantities of lipids to WTs, TRPV1KOs fed HFD gain less weight, present less adiposity and greater thermogenesis [241]. On the other hand, in ageing mice fed HFD, the lack of TRPV1 promotes obesity due to altered energy balance and leptin resistance [219]. In another study with mice given HFD, no differences were noted between WT and TRPV1KO mice in regards to weight gain and adipose tissue mass [173]. It is possible that the differences between these studies are due to variations in the fat contents of HFD. Irrespective of this controversy, the above evidence indicates a promising clinical use of TRPV1 agonists such as capsaicin to preventing obesity by activating TRPV1. In agreement, capsaicin intake increases lipolysis in exercising individuals [242]. 

TRPV1 is not the only TRP channel to modulate thermogenesis. In this context, the alkamide trans-pellitorine found in *Piper nigrum* (black pepper) impairs lipid accumulation by reducing PPARγ levels in 3T3-L1 cells during the differentiation and maturation phases via the indirect activation of TRPV1 and TRPA1 [243]. This indicates a synergistic contribution of the functional expression of both channels in the regulation of energy expenditure. Corroborating these findings, the incubation of cinnamaldehyde diminished TG and phospholipid content in 3T3-L1 preadipocytes by down-regulating PPARγ expression and increasing AMP-activated protein kinase levels [244,245]. TRPA1-independent pathways of thermogenesis and metabolic reprogramming were also reported for cinnamaldehyde; the compound was shown to promote these responses in mouse and human adipose cells by increasing UCP-1 and SOD expressions [245]. 

It is also possible that the neuronal expression of TRPA 1, probably in the vagus nerve, contributes to thermogenesis as the receptor agonists cinnamaldehyde and allyl isothiocyanate, both induce adrenaline secretion and prevent fat accumulation and obesity in rats [246]. The same study showed the ability of cinnamaldehyde to activate BAT and reduce visceral fat in animals fed high-fat/high-sucrose diet. Supporting data demonstrated that cinnamaldehyde decreases weight gain, and the quantities of plasma TG, non-esterified fatty acid, and cholesterol in mice with HFD [247], and also that incubation of cinnamaldehyde with 3T3-L1 cells decreases TG and phospholipid accumulation, whilst reducing PPARγ; these effects were blocked by the TRPA1 antagonist AP-18 [244]

The contribution of TRPA1 activation to thermogenesis has been supported not only by studies with exogenous agonists such as cinnamaldehyde, but also by those performed with endogenous activators of the channel including 4-HNE. High levels of 4-HNE were detected in the subcutaneous adipose tissue of obese subjects [248]. Incubation of 4-HNE with subcutaneous adipocytes triggered the production of ROS (H_2_O_2_) and antioxidant enzymes (TRX, SOD and catalase), associated with reduced growth and differentiation of preadipocytes [248]. The down-regulation of adiponectin by 4-HNE has been previously discussed, and it is known to occur by degradation of adiponectin protein following incubation with the aldehyde via the ubiquitin-proteasome system [249]. Of note, although 4-HNE reduces adipogenesis, its inhibitory effects on adiponectin may reflect in inflammation, and worsening of MS. Indeed, 4-HNE induced TNFα gene transcription in WAT samples of obese subjects [250]. Despite the interesting actions of cinnamaldehyde and 4-HNE in adipogenesis, the specific contributions to TRPA1 activation in this response is yet to be established by further studies employing strategies including KO mice, knockdown and antagonists for the channel. 

TRPC1/TRPC5 complexes were also identified in cultured 3T3-L1 cells and in perivascular adipose tissue samples obtained from mice and humans [147,200]. The constitutive activation of these complexes in the mature adipocytes of the perivascular fat was suggested to act as a negative regulator of adiponectin [147]. In vitro TRPC1/TRPC5 knockdown increased adiponectin generation in mice, disruption of TRPC5-containing complexes and enhanced adiponectin levels irrespective of the diet composition (chow or HFD) [147]. Interestingly, the same study showed that the inhibitory effects of TRPC1/TRPC5 complexes on adiponectin were halted by exposure to dietary ω-3 fatty acids in differentiated 3T3-L1 cells.

### 4.3. TRPs and the Liver

Several reports show that TRPs are also relevant for the reestablishment of liver function during MS. From a therapeutic point of view, the improvement of mitochondrial metabolism is a pertinent strategy aimed for the treatment of non-alcoholic fatty liver disease (NAFLD), as enhanced hepatic oxidative stress is correlated with inflammation in obesogenic diets [251]. In this case, TRPV1 activation secondary to the dietary intake of low-dose capsaicin prevented the hepatic damage observed in NAFLD via uncoupling protein 2 (UCP-2) up-regulation in mice [127,252]. Li and collaborators [127] described TRPV1 expression on hepatocytes and the mechanisms triggered by its activation. The observed effects comprise reduced lipid accumulation and TG concentrations levels in WT, but not in TRPV1KO animals [127]. UCP-2 up-regulation secondary to TRPV1 activation was also associated with other therapeutic effects, such as the reversal of hyperglycaemia-induced endothelial dysfunction in mice. Such an antioxidant mechanism via UCP-2 may be a multifaceted link between the dietary intake of capsaicin and its therapeutic effects in either metabolic or cardiovascular diseases [253]. 

Other liver functions may also benefit from low-dose dietary intake of capsaicin, such as lipoprotein metabolism. Although it was demonstrated that capsaicin does not reduce oxLDL accumulation in TNFα sensitized macrophages, TRPV1 activation up-regulated ATP-binding cassette (ABCA1 and ABCG1) expression via liver X receptor α, thus enhancing cholesterol efflux from the cells [254]. These findings are also relevant in the physiopathology of atherosclerosis, as oxLDL is a widely known biomarker of both atherosclerosis and NAFLD [253,254].

As demonstrated in mice receiving chronic dietary capsaicin, reduced inflammatory biomarkers and up-regulation of PPARδ secondary to TRPV1 activation takes place in WT but not in TRPV1KO animals with NAFLD [255]. Noteworthy, TRPV1 plays a substantial role in the obesity pathogenesis, with important consequences for hepatic health. TRPV1KO mice demonstrated a more pronounced hepatic steatosis when fed HFD, which was correlated with reduced expression of PPARα and oxidation of fatty acids. In addition, the impaired glucose metabolism and hepatic health observed in TRPV1KO mice are some of the evidence confirming the significant relationship between TRPs and MS-related diseases, as recently described by Baskaran and collaborators [256].

However, the role of TRPV1 in other hepatic diseases may be in contrast with the results so far, thus evidencing the complexity of this matter. For example, the genetic depletion of TRPV1 did not blunt hepatic steatosis but prevented the hepatic injury in chronic alcoholic hepatic disease [257], thus evidencing different roles for TRPV1 in the pathogenesis of different hepatic diseases and making clear that TRPV1 activation is not an obvious pathway to be clinically explored, mainly in the case of MS patients with other comorbidities.

The effects of the cinnamaldehyde have been investigated in the liver of T2D and gestational diabetic rats induced by high fat/high sucrose diet [258,259]. Intragastric cinnamaldehyde treatment significantly decreased hepatic lipid peroxidation, steatosis and inflammation, and enhanced hepatic GSH and SOD levels in rats with T2D. These changes were associated with enhanced insulin sensitivity [258]. In addition, the oral administration of cinnamaldehyde controlled hyperphagia and glucose intolerance in rats with gestational diabetes [259]; such effects were associated with reduced circulating levels of total cholesterol, triglycerides, leptin and TNFα, and higher levels of high-density lipoprotein (HDL)-cholesterol, adiponectin, liver glycogen and PPARγ expression, and the activity of antioxidant enzymes. On the other hand, analysis of healthy human liver samples by in situ hybridization demonstrated the expression of TRPA1 in the sinusoidal endothelial lining and Kupffer cells, but not in hepatocytes [260]. Thus, if cinnamaldehyde effects are due to TRPA1 activation, this would occur via endothelial and/or Kupffer cells, and this is yet to be confirmed by future research.

So far, there is limited information on the expression of TRPC sub-family members in the liver [208,260]; however, the current data do not support a role for TRPC5 in the liver in MS. Nonetheless, TRPC5 was found to mediate cholestasis in mice, as TRPC5KOs protected against the disease once they presented attenuated liver enlargement, reduced hepatic bile acid and lipid content, diminished liver enzymes, and decreased hepatic cholesterol, TG and phospholipid contents [261].

### 4.4. TRPs and Skeletal Muscle

The importance of the skeletal muscle to metabolic syndrome has been well documented and discussed [262,263]. Skeletal muscle is considered the largest tissue of the body sensitive to insulin, and it is where most of the insulin-mediated glucose uptake by GLUT4 occurs [264]. This tissue is also a producer of myokines which include cytokines (IL-6), myostatin, myonectin, irisin, and musclin [265]. These are released during muscle contraction, during exercise for example [266,267], and have endocrine and paracrine functions acting in other metabolic organs (liver, adipose tissue, and pancreas).

TRPV1 expression was first described in the rat skeletal muscle sarcoplasmic reticulum [202] and it was later confirmed in the human tissue as a target for endocannabinoids [203]. The latter finding indicated that TRPV1 mediates the down-regulatory effects of these molecules on adiposity. This was supported by data from skeletal L6-cells in which the TRPV1 antagonist SB-366791 blocked the insulin-induced glucose uptake triggered by the endocannabinoid 2-arachidonoylglycerol [204].

In another study, functional TRPV1 was detected in mouse myocytes (C2C12 cells) and skeletal muscle [268]. Indeed, the in vitro incubation of capsaicin triggered Ca^2+^ influx, and increased glucose oxidation and ATP production in C2C12 cells; both responses were blocked by TRPV1 antagonists (5′-iodo-resiniferatoxin-α or SB-452533) [268,269]. Of note, glucose oxidation and ATP generation in C2C12 cells were suggested to happen independent of insulin [269]. In another report, capsaicin induced the up-regulation of TRPV1 and peroxisome proliferator-activated receptor-γ coactivator-1α (a regulator of lipid and glucose metabolism, mitochondrial biogenesis and muscle remodelling in myocytes, and enhanced mitochondrial biogenesis and ATP production in myotubes [268,270]. Analysis of the gastrocnemius muscle indicated that the myocytes of mice fed with z capsaicin-supplemented diet exhibited a similar phenotype to that observed in vitro [268]. In addition, the same study showed that capsaicin enhances exercise endurance whilst lowering the levels of blood lactic acid and TGs in WT but not TRPV1KO mice; similar data were gathered from mice over-expressing the receptor which also presented with greater numbers of oxidative muscle fibres. In another study, TRPV1KO mice fed HFD presented higher insulin resistance in WAT and BAT, but not in the skeletal muscle in comparison to WTs [219]. Overall, the results suggest that the activation of skeletal muscle-located TRPV1 contributes towards thermogenesis and enhanced insulin sensitivity; both responses are exacerbated by exercise.

Functional TRPA1 was identified in primary human myoblasts but became down-regulated during differentiation to skeletal muscle cells [207]. Indeed, TRPA1 agonists such as allyl isothiocyanate induced Ca^2+^ currents in these cells that were blocked by the TRPA1 antagonists HC-030031 and A967079. The same study demonstrated that TRPA1 activation causes myoblast migration and fusion, and suggested this receptor is an important sensor of muscle damage and inflammation and, therefore, contributes to muscle repair.

A functional role in the maintenance of skeletal muscle force during sustained repeated contractions was shown for TRPC1 [271]. TRPC1 activation also annuls the beneficial effects of exercise on obesity-associated T2Din mice [233]. In addition, the activation of TRPC1/TRPC4 complexes is key to myogenesis and skeletal muscle differentiation [272]. On the other hand, the expression of TRPC5 and its function in the skeletal muscle is controversial. In fact, there is conflicting data on its expression on skeletal myoblasts [271,273]. Therefore, the possible roles of TRPC5 in MS via the skeletal muscle remain and deserve to be investigated.

### 4.5. Connecting Metabolic Tissues and the Central Nervous System

The CNS has an important role in the regulation of food intake and energy metabolism. After a meal, satiation signals are sent by the gastrointestinal tract to multiple centres in the CNS (hypothalamus and the brainstem), as well as adiposity signals about energy availability in the WAT. Then, humoral and neuronal outputs are sent from the CNS to the peripheral metabolic tissues in order to regulate energy metabolism. These aspects have been previously reviewed and discussed [274,275]. Herein, we present the current data that connect the CNS to the periphery in the regulation of energy metabolism via TRPs.

Evidence indicates that TPRV1 interacts with the CNS via appetite regulating hormones such as ghrelin (an orexigenic peptide found in the stomach [275], leptin, and the glucagon-like peptide-1 (GLP-1; an anorexigenic peptide hormone secreted by intestinal L-cells and pancreatic α-cells, and the brain [275,276,277]). Human data indicate that the acute TRPV1 activation increases GLP-1 and diminishes ghrelin levels in the plasma samples of individuals receiving a capsaicin-containing meal, as soon as 15 min after consumption, without altering energy expenditure [278]. Capsaicin effects on satiety are controversial with some studies indicating the compound reduces energy intake [279,280] and others showing no effects [278,281].

The stomach, especially the pyloric portion and duodenum, and the small and large intestines, express functional TRPA1 [282]. In the stomach, TRPA1 is expressed in ghrelin-producing cells. TRPA1 expression was also shown in the MGN3-1 cell line; this, when incubated with cinnamaldehyde, presents up-regulation of TRPA1 and insulin receptor mRNAs and reduced secretion of ghrelin. Cinnamaldehyde effects on ghrelin secretion were partially attenuated by TRPA1 antagonism with HC-030031. In vivo, the acute oral administration of cinnamaldehyde caused reduction in food intake in the initial 2h following treatment and delayed gastric emptying in WTs but not TRPA1KO mice. Repeated treatment with the compound did not affect food intake, but reduced body weights and fat mass, and improved insulin sensitivity in mice fed HDF [282]. The same mice presented increased expression of glucose transporters and of genes involved in fatty acid oxidation in WAT and BAT. TRPA1 involvement in ghrelin production was confirmed by another study in which intragastric β-eudesmol, an oxygenized sesquiterpene, increased food intake and plasma octanoyl ghrelin levels [283]. β-eudesmol also enhanced gastric vagal nerve activity, a response diminished by different TRPA1 antagonists and deletion of TRPA1 receptor. Despite the conflicting results, the data show that TRPA1 regulates ghrelin secretion and food intake; however, the degree of regulation may depend on the TRPA1 agonist and the activated pathways.

Additionally, TRPV1 is functionally expressed on the intestinal cell line secretin tumour cell-1 (STC-1) and in mouse ileum samples known to produce GLP-1 [284]. In the intestinal cells, capsaicin stimulated the production of GLP-1 which was blocked by the TRPV1 antagonists capsazepine and 5′-iodo-resiniferatoxin-α. Intragastric capsaicin increased plasma GLP-1 levels following glucose challenge in WTs and in mice with T2D, a response impaired by treatment with 5′-iodo-resiniferatoxin-α or receptor ablation [284]. Hypothalamic pro-opiomelanocortin neurones are involved in food intake and express functional TRPV1 [205]. These neurones respond to GLP-1 release via the GLP-1 receptor, and are also the site of action of liraglutide, a GLP-1 analogue used in the treatment of T2D [285]. In a recent report, GLP-1 was suggested to activate TRPV1/TRPA1-dependent Ca^2+^ currents in GLP-1 receptor-expressing enteric neurones, and the subsequent release of substance P [286]. It is possible, therefore, that GLP-1 may elicit Ca^2+^ influx via TRPs in hypothalamic pro-opiomelanocortin neurones.

Mouse intestinal L cells and the small intestine express functional TRPA1, which responds to allyl isothiocyanate and polyunsaturated fatty acids in vitro [287]. Indeed, the Ca^2+^ currents elicited by these compounds were blocked by the TRPA1 agonist A-967079. Allyl isothiocyanate caused GLP-1 release from intestinal cells in a TRPA1-dependent manner, without altering glucose-induced secretion of GLP-1. GLP-1 secretion was abolished in TRPA1KO intestinal cells and in those treated with HC-033031 [288]. Additionally, TRPA1 was found to mediate AS1269574-induced GLP-1 production in intestinal cells (STC-1 cells) [288]. Noteworthy, AS1269574 is an agonist of G protein-coupled receptor 119 (GPR119), an important enteroendocrine sensor of dietary triglyceride metabolites expressed in intestinal cells. Glucagon production triggered by AS1269574 though, is a direct result of GPR119 activation, with no involvement of TRPA1 [223]. The non-electrophilic small molecule GLP-1 secretagogue JWU-A021 produced TRPA1-dependent Ca^2+^ currents in STC-1 and primary intestinal cells, which were suppressed by the antagonists A967079 and HC030031 [223]. More recently, allicin, another dietary TRPA1 agonist, restored GLP-1 levels and insulin sensitivity in HFD-fed mice [289]. These data indicate that intestinal located TRPA1 mediates GLP-1 release.

Leptin activates its receptor on hypothalamic pro-opiomelanocortin neurones and causes the subsequent increase in the levels of the anorectic peptide α-melanocyte-stimulating hormone, whilst inhibiting neuropeptide Y (NPY) neurones [290,291,292]. High levels of this hormone are present in most obese subjects and animals [293,294]. This is suggested to be due to the necessity for high circulating levels of leptin to overcome resistance to its action and maintain energy homeostasis [295]. Leptin resistance and altered energy balance have been attributed to obesity in TRPV1-null mice fed HFD [219]. Treatment with leptin did not reduce food intake, and leptin-mediated hypothalamic signals were impaired in the TRPV1KO mice [219]. These animals were more obese and insulin-resistant than their counterparts. On the other hand, in another study, leptin levels were raised in both TRPV1 WTs and KOs [173]. TRPV1 activation also enhanced the frequency of miniature excitatory synaptic currents in leptin receptor-containing neurones in stomach-associated brainstem dorsal motor nucleus of the vagus [296]. Evidence also indicates that TRPV1 receptor activity is diminished in the brainstem dorsal vagal complex of diabetic mice [297]. These data suggest that TRPV1 mediates the effects of leptin.

No reports have linked TRPA1 activation/expression to leptin signalling and its connection to the brain regions involved in hunger and energy expenditure. On the contrary, TRPC5 has been indicated as an interesting target to regulating leptin responses. In fact, the neuronal deficiency of TRPC5 or its deletion in pro-opiomelanocortin neurones leads to obesity associated with decreased energy expenditure and higher food intake in mice [209]. The same study demonstrated that both leptin and serotonin 2C receptor-agonists exert their acute anorexigenic effects via TRPC5 activation. TRPC5 complexes also contribute to melanocortin neuronal activity, thus altering energy metabolism and feeding behaviour [209]. Moreover, the intracerebroventricular injection of insulin resulted in a similar response of energy expenditure via TRPC5 activation [298]. Both insulin and leptin were suggested to activate TRPC5 indirectly, following their binding to their specific receptors and downstream signalling (phosphatidylinositide-3 kinase and phospholipase Cγ activation) [298]. Since both TRPC1 and TRPC4 are functionally expressed in pro-opiomelanocortin neurones [298], it is possible that all TRPC5 complexes contribute to the metabolic responses mediated by these cells. The protective role of neuronal TRPC5 complexes in MS is supported by data obtained from studies on GLP-1 agonists and their effects on pro-opiomelanocortin neurones [210,211]. Indeed, both liraglutide and semaglutide actions on pro-opiomelanocortin neurones involve TRPC5 activation in vivo and in mouse hypothalamic slices. Of note, in the hippocampus, leptin-dependent responses do not require TRPC5 expression [299].

Interestingly, mitochondrial-derived ROS are produced by brain neuronal cells of different regions including the hypothalamus [300,301] and are involved in central glucose [302] and hypertriglyceridemia sensing [303]. Accordingly, H_2_O_2_ causes a marked increase in the firing of hypothalamic pro-opiomelanocortin neurones and decreased feeding in mice [304]. Considering the ability of TRP channels to sense this ROS, it is also possible they mediate ROS signalling in these neurones.

## 5. Clinical Perspectives

In addition to non-clinical studies, the beneficial effects of modulating TRPV1, TRPA1 and TRPC5 channels in obesity, T2D, atherosclerosis and MS have been investigated in a range of clinical trials.

In these, pungent and non-pungent activators of TRPV1 have been assessed. In a study, either 0.25% capsaicin or placebo were given to 24 subjects (12 men and 12 women) with body mass index (BMI) of 25.0, 30 min before meal. Oral capsaicin enhanced satiety and diminished calorie and fat intake [279]. In another report with 19 overweight to obese men, a supplement containing capsaicin increased energy expenditure in comparison to placebo [305]; these findings were supported by further studies [306,307,308,309,310]. The use of capsaicin 1h prior to low intensity exercise was also shown to improve lipolysis in healthy volunteers [242]. Moreover, capsaicin from *Capsicum frutescens* had hypoglycaemic effects in healthy individuals [311]. Capsinoids are non-pungent capsaicin-related substances [312]. Individuals with BMI between 25.0 and 35.0 received capsinoid oil (6 mg/day) obtained from *Capsicum anuum* L. variety CH-19 Sweet or placebo, capsinoids decreased body weight whilst enhancing fat oxidation [313]. The same study found a correlation between reduction of abdominal fat and the genetic variants TRPV1 Val585Ile and UCP-2-866 G/A. Another capsinoid, dihydrocapsiate, caused a small thermogenic effect in healthy subjects [314]. Consumption of a supplement containing low dose capsinoids (2 mg) led to increased plasma levels of FFA [315]. Accordingly, *C. anuum* capsinoids increased energy expenditure by activating BAT in healthy subjects in comparison with the placebo group [316,317]. The above data indicate the potential of capsaicin to treat obesity and hyperglycaemia. On the other hand, although promising, the thermogenic effects of capsinoids are yet to be confirmed in further studies with overweight and obese individuals.

Cinnamaldehyde is a major compound found in cinnamon barks [318]. Several studies have investigated the beneficial effects of cinnamon in T2D. In a study with 60 T2D subjects (30 woman and 30 men), intake of cinnamon capsules attenuated serum glucose, TG, total and low-density lipoprotein cholesterol [319]. In agreement, cinnamon extracts or supplements decreased plasma glucose levels and malondialdehyde concentrations, and improved lipid profile in overweight to obese individuals [320,321,322,323], and induced hypoglycaemia in T2D patients [324] and healthy subjects [325]. Conversely, cinnamon powder or supplement consumption did not alter plasma glucose or serum lipid profile in T2D patients [326,327]. A similar result was observed in postmenopausal patients with T2D [328] and healthy individuals [329]. Interestingly, cinnamon powder intake lowered blood pressure and glycated haemoglobin in patients with T2D [330]. Although the evidence gathered from these studies are controversial, they raise attention for further studies to support the potential use of cinnamon and derived compounds in the management of MS.

No clinical trials assessing the impact of TRPC5 in human MS have been published to date. Nonetheless, the use of TRPC4/TRPC5 inhibitors for cosmetic weight loss as well as to combat obesity, T2D, MS, NAFLD and non-alcoholic steatohepatitis was recently published (accession numbers: WO/2018/146485; EP3579838; US20200345741). Liraglutide and semaglutide antidiabetic actions in the hypothalamus require TRPC5, which indicates this channel is an interesting target for the development of novel therapies for MS.

Nonetheless, considering that MS is a complex disease, it is not surprising that other TRPs, in addition to TRPV1, TRPA1 and TRPC5, may influence the balance between oxidative stress and inflammation during disease progression. For instance, TRPM2 is another TRP activated by ROS (specifically, H_2_O_2_), which is involved in insulin resistance [213,331]. TRPM4 and TRPM5 expression were also described in human Langerhans islets, further indicating possible roles with clinical perspectives for MS [332]. Of note, TRPM2 expression is significantly enhanced during NAFLD, and its activation by ROS overproduced during the disease plays a significant role in pathophysiology contributing to its progression [333]. Natural antioxidants such as saliroside (from *Rhodiola rosea*) and curcumin are both able to inhibit TRPM2 activation in hepatocytes, resulting in reduction of lipid deposition, diminished expression of cytokines (IL-1β and IL-6) and protection against cell damage [333,334]. These are early findings and further research in the field is important and deserves to be pursued.

## 6. Conclusions

Most of the metabolic alterations comprised in MS are correlated with altered expression of TRPs and are directly connected with the observed vascular dysfunction in T2D and obesity. Herein, the available information on the contribution of TRPV1, TRPA1 and TRPC5 to MS is discussed and summarized in Table 3.

Overall, these channels are involved in the regulation of different pathways of MS, including hormone production, inflammation, and ROS generation at systemic levels and different metabolic tissues (adipose, pancreatic, hepatic and skeletal muscle), connecting those to the CNS. The different patterns of expression of these channels across tissues confer on them the ability to control a variety of cell functions. Non-clinical and clinical data clearly highlight the potential of ligands for these channels, especially natural compounds such as capsaicin/capsinoids and cinnamaldehyde, to treating the various aspects of MS, from insulin resistance to atherosclerosis. Considering the multiple mechanisms underlying MS establishment and progression, it is possible that a combination of TRP ligands may confer better control of adiponectin release, ROS production, and inflammation in the disease. In this context, the dual roles of TRPs such as that of TRPA1 in atherosclerosis must be considered.

## Figures and Tables

**Table 1:** Evidence for the functional activation of TRPV1, TRPA1 and TRPC5 by reactive molecules involved in metabolic syndrome.

TRP Channel	Reactive Molecule	Cell Type	Activation Mode	Ca^2+^ Influx	Electrophysiology
TRPV1	H_2_O_2_	HEK293T [123,150,152]	Sensitization	✓	✓
		Bovine aortic endothelial cells [123]	Sensitization	✓	
TRPA1	H_2_O_2_	HEK293T [157,158,159]	Direct	✓	
		DRG neurones [158,159,160]	Direct	✓	
		Bladder neuronal afferents [161]	Direct		✓
		CHO cells [160]	Direct	✓	
	NO	HEK293T [159]	Direct	✓	
		DRG neurones [159]	Direct	✓	
	H+	HEK293T [159]	Direct	✓	
		DRG neurones [159]	Direct	✓	
	Aldehydes (4-HNE and 4-ONE)	HEK293T cells [157]	Direct	✓	
		DRG and trigeminal ganglia neurones [157,160]	Direct	✓	
		CHO cells [160,162]	Direct	✓	
TRPC5	H_2_O_2_	HEK293T cells [145]	Direct	✓	
	Reduced TRX	HEK293T cells [150]	Direct	✓	
		Synoviocytes [150]	Direct	✓	

**Table 2:** Evidence for TRPV1, TRPA1 and TRPC5 expression on cells and in tissues involved in metabolic syndrome.

TRP Channel	Cell/Tissue	PCR/ qPCR	Immunostaining/ Immunofluorescence	Western Blot	Ca^2+^ Influx	Electrophysiology
TRPV1	adipose tissue/adipocytes [124,125,200,201]	✓	✓	✓	✓	
	liver [126,127]	✓	✓	✓	✓	
	M1 macrophages [128]		✓		✓	
	pancreatic β-cells/langerhans islets [132]	✓	✓	✓		
	coronary endothelial cells [123]					✓
	T cells [129,130,131]		✓	✓	✓	✓
	skeletal muscle [202,203,204]	✓	✓	✓	✓	
	pro-opiomelanocortin neurones [205]	✓	✓	✓		✓
TRPA1	pancreatic β-cells/langerhans islets [137,206]	✓	✓	✓	✓	✓
	T cells [175,176,177]	✓	✓	✓	✓	✓
	skeletal muscle cells [207]	✓	✓	✓	✓	✓
	monocytes/macrophages [179,180]	✓	✓	✓	✓	
TRPC5	endothelial cells [166]				✓	
	T cells [185]	✓			✓	
	M1 macrophages [186]	✓		✓		
	pancreas [208]	✓				
	adipose tissue [147,200]	✓	✓	✓	✓	✓
	pro-opiomelanocortin neurones [209,210,211]					✓

**Table 3:** Overall contribution of TRPV1, TRPA1 and TRPC5 to metabolic syndrome: a summary of endogenous agonists, expression sites and roles.

TRP Channel	Endogenous Agonists	Expression Site	Role in MS
TRPV1	12 (S)-HPETE [118], 20-HETE [119], 9-HODE and 13-HODE [120], anandamide [121], H_2_S [122], ROS (H_2_O_2_) [123]	Adipose tissue/adipocytes [124,125,200,201], liver [126,127], M1 macrophages [128], pancreatic β-cells/langerhans islets [132], coronary endothelial cells [123], T cells [129,130,131], skeletal muscle [202,203,204], pro-opiomelanocortin neurones [205]	Increase of insulin sensitivity [216,220,221], browning of WAT, reduction of lipid synthesis and obesity/adiposity [201,203,204,239], enhanced thermogenesis [240] and leptin sensitivity [219], reduction of lipid accumulation and TG [127], protection against endothelial dysfunction [253], increase of GLP-1 and attenuation of ghrelin production [278]
TRPA1	Methylglyoxal [138], 4-HNE, 15-deoxy-delta(12,14)-prosta-glandin J_2_ (15d-PGJ_2_) and H_2_O_2_ [139]	Pancreatic β-cells/langerhans islets [137,206], T cells [175,176,177], adipocytes [244,245], vagus nerve [246]	Macrophage-mediate responses in atherosclerosis [180,184], increase of insulin secretion [137,222,223,224,225] and sensitivity [228,258,259], reduction of insulin signalling and insulin-induced glucose uptake in skeletal muscle cells [229], weight loss and reduction of TG and cholesterol [244,247], attenuated adipogenesis [250], increased adipose tissue inflammation and ROS [248,250] reduction of ghrelin [282], production of ghrelin [288]
TRPC5	H_2_O_2_ [145], reduced TRX [146], and fatty acids [147]	Endothelial cells [166], T cells [185], M1 macrophages [186], pancreas [208], adipose tissue [147,200], pro-opiomelanocortin neurones [209,210,211]	Polarization of macrophages to M2 and protection against atherosclerosis [186], negative regulation of adiponectin [147], enhance of energy expenditure [209,298]

## Data Availability

Not applicable.
